# STAT5 triggers *BCR-ABL1* mutation by mediating ROS production in chronic myeloid leukaemia

**DOI:** 10.18632/oncotarget.806

**Published:** 2012-12-31

**Authors:** Wolfgang Warsch, Eva Grundschober, Angelika Berger, Lars Gille, Sabine Cerny-Reiterer, Anca-Sarmiza Tigan, Andrea Hoelbl-Kovacic, Peter Valent, Richard Moriggl, Veronika Sexl

**Affiliations:** ^1^ Institute of Pharmacology and Toxicology, Veterinary University Vienna, Veterinaerplatz 1, 1210 Vienna, Austria; ^2^ Department of Internal Medicine I, Division of Hematology & Hemostaseology, Medical University of Vienna (MUV), Austria; ^3^ Ludwig Boltzmann Cluster Oncology, Vienna, Austria; ^4^ Ludwig Boltzmann Institute for Cancer Research (LBI-CR), Vienna, Austria

**Keywords:** BCR-ABL1, Chronic Myeloid Leukaemia, Reactive Oxygen Species, STAT5, mutation

## Abstract

We recently reported that chronic myeloid leukaemia (CML) patients harbour high levels of STAT5 when they progress to advanced phases of disease. Advanced disease is characterized by an increased incidence of *BCR-ABL1* mutations. We now describe a highly significant correlation between STAT5 expression and the incidence of *BCR-ABL1* mutations in primary CML. Forced expression of STAT5 in murine BCR-ABL1 transformed cells sufficed to enhance the production of reactive oxygen species (ROS) and to trigger DNA damage. STAT5-mediated ROS production is independent of JAK2 but requires concomitant BCR-ABL1 signalling as forced STAT5 expression in untransformed *BCR-ABL1* negative cells has no impact on ROS levels. Only within the context of a *BCR-ABL1* positive cell does STAT5 transcriptionally regulate a target gene or set of genes that causes the enhanced ROS production. Our study suggests the existence of a feed-forward loop accelerating disease progression, in which BCR-ABL1 enhances its own mutation rate in a STAT5-ROS dependent manner. This model explains the increased occurrence of inhibitor-resistant *BCR-ABL1* mutations in advanced disease stages driven and characterized by high STAT5 expression.

## INTRODUCTION

The *BCR-ABL1* oncogene results from the t(9;22)(q34;q11) reciprocal translocation generating the Philadelphia chromosome (Ph). *BCR-ABL1*^+^ CML is a stem cell-derived disease that progresses in three distinct phases: chronic phase (CP), which may last for several years, accelerated phase (AP), and finally blast crisis (BC) that is refractory to therapy [1]. The tyrosine kinase inhibitor (TKI) imatinib revolutionized the treatment of *BCR-ABL1*^+^ leukaemia being the first drug successfully targeting an oncogenic tyrosine kinase [2]. Imatinib paved the way for this group of signal interceptors and the TKIs imatinib, nilotinib and dasatinib are now used as first-line therapy in CP-CML patients [3-5]. Although these substances are highly effective in eradicating the vast majority of peripheral CML cells, residual *BCR-ABL1*^+^ progenitors persist in CML patients despite undetectable molecular disease and may only be eliminated after continuous treatment for several years [6-8]. In addition, some patients fail to respond to BCR-ABL1 TKIs (primary resistance) or relapse after an initial promising response (secondary resistance). Secondary resistance is most frequently caused by point mutations in the *BCR-ABL1* kinase domain that render the kinase insensitive to TKI treatment [9, 10]. Imatinib resistance frequently represents the first signal that the disease progresses into a more advanced stage to accelerated phase and finally terminal blast crisis associated with short median survival times [11].

Genomic instability is one of the hallmarks of a transformed cell and provides the basis for the development of *BCR-ABL1* mutations under the selection pressure of TKI therapy [12]. Genomic instability may result from increased DNA damage as well as an aberrant DNA repair machinery [13]. DNA damage may be caused by external triggers including UV-light, radiation, and certain chemicals. The production of endogenous reactive oxygen species (ROS) – a cell intrinsic process - is another well described inducer of DNA damage. Several studies have defined ROS - especially hydroxyl radicals (HO*) and lipid peroxidation products - as major sources for Amutations in various forms of cancer [14, 15]. The potential of ROS to induce spontaneous DNA double-strand breaks (DSBs) combined with impaired DNA repair responses, make ROS a potent trigger/inducer of chromosomal aberrations [16-19].

BCR-ABL1 activity has convincingly been linked to ROS production [20]. BCR-ABL1 induced ROS production combined with impaired DNA damage responses (mainly via RAD51 de-regulation) is thought to promote genomic instability and finally self-mutagenesis; a process which has the potential to provoke TKI resistance [11, 17, 18].

Aside from BCR-ABL1 expression, the pronounced activation of the transcription factor STAT5 is considered a signalling hallmark in CML cells [21-23]. STAT5 signalling is crucial for transformation by BCR-ABL1 and thus represents a bottle neck for disease induction. Importantly – as critical for therapeutic intervention and defining STAT5 as drug target - STAT5 is also essential for leukaemia maintenance [24-29]. The importance of STAT5 in CML pathogenesis is further underscored by the fact that STAT5 expression increases during disease progression and that high STAT5 levels significantly decrease imatinib sensitivity [30, 31].

In this study we investigated the potential connection between the transcription factor STAT5 and the occurrence of *BCR-ABL1* mutations. We describe the highly significant correlation between STAT5 expression levels and the frequency of *BCR-ABL1* mutations in human CML patients. We propose a model where STAT5 triggers ROS production and thereby induces DNA damage. The concomitant STAT5 dependent up-regulation of anti-apoptotic genes enables the cells to survive and allows for the development of *BCR-ABL1* mutations thereby explaining the increased occurrence of inhibitor-resistance in advanced disease stages.

## RESULTS

### Elevated STAT5A mRNA levels correlate with BCR-ABL1 mutation rates

We have recently shown that STAT5 expression levels increase during CML progression and that elevated activated STAT5 levels account for a reduced responsiveness to BCR-ABL1 tyrosine kinase inhibitor (TKI) therapy [30]. The STAT5 mediated TKI resistance was independent of *BCR-ABL1* mutations or *BCR-ABL1* expression levels but critically dependent on an intact STAT5 signalling. The initiative for our current study came from an experiment where p160^v-ABL^ transformed murine cells infected with a retrovirus encoding for *Stat5a-IRES-GFP* (STAT5A) displayed enhanced colony formation over an empty vector (GFP) control when plated in methylcellulose containing 1 μM imatinib. Three weeks thereafter, clones were visible in the dishes containing cells ectopically expressing STAT5A (Supporting Information Fig S1A) whereas hardly any colony was visible in the control cultures. Similar observations were made with Ba/F3*p210^BCR-ABL1^* cells that were seeded in 96 well plates in medium enriched with 2 μM imatinib. Ba/F3*p210^BCR-ABL1^* cells ectopically expressing STAT5A gave rise to more imatinib-resistant clones compared to control cells (Supporting information Fig S1B). Hence, even under conditions where we continuously blocked BCR-ABL1 activity using high imatinib concentrations, STAT5 expression conferred an advantage and allowed some cells to escape BCR-ABL1 TKI therapy. One frequent cause for imatinib resistance is the occurrence of mutations within the *BCR-ABL1* oncogene. To test a potential correlation between STAT5 expression levels and the frequency of *BCR-ABL1* mutations we decided to analyse leukemic samples from a cohort of 50 CML patients including our initial patient sample collection [30] (For patient characteristics see Table 1).

**Table 1:** Part A Patient characteristics; WBC, white blood cell count; PB, peripheral blood; BM, bone marrow; IS, international standard

Patient #	CML Phase	WBC	PB% blasts	BM % blasts	% basophiles	Thrombo cytes	*BCR-ABLI* mutation	IS value
1	CP	23.67	0	1	1	209	non	49.809
2	CP	n.d.	n.d.	n.d.	n.d.	n.d.	non	n.d.
3	CP	n.d.	n.d.	n.d.	n.d.	n.d.	non	n.d.
4	CP	34.48	n.d.	<1	1	145	non	35.153
5	CP	142.1	6	3	6	822	non	53.96
6	CP	208	2	1	1	373	non	83.53
7	CP	181.87	1	2	4	148	non	53.441
8	CP	6.25	n.d.	n.d.	13	227	non	83.53
9	CP	30.4	1	<1	19	11	non	45.389
10	CP	n.d.	n.d.	n.d.	n.d.	n.d.	non	n.d.
11	CP	n.d.	n.d.	n.d.	n.d.	n.d.	non	n.d.
12	CP	32.6	2	1	5	326	non	25.609
13	CP	216.4	5	3	5	574	non	57.38
14	CP	271	n.d.	n.d.	n.d.	n.d.	non	n.d.
15	CP	38.39	3	n.d.	5	311	non	58.85
16	CP	258.48	0	n.d.	7	622	non	41.28
17	CP	34.22	0	n.d.	11	911	non	60.021
18	CP	127.7	1	<1	1	93	non	58.652
19	CP	23.67	0	1	1	209	non	n.d.
20	CP	35.94	0	n.d.	2	186	non	59.399
21	CP	216.4	5	3	5	574	non	n.d.
22	CP	53.8	2	n.d.	2	186	non	50.172
23	CP	n.d.	n.d.	n.d.	n.d.	n.d.	non	55.866
24	CP	n.d.	n.d.	n.d.	n.d.	n.d.	non	54.344
25	CP	n.d.	n.d.	n.d.	n.d.	n.d.	non	48.487
26	CP	n.d.	n.d.	n.d.	n.d.	n.d.	non	45.434
27	CP	n.d.	n.d.	n.d.	n.d.	n.d.	non	68.857
28	CP	n.d.	n.d.	n.d.	n.d.	n.d.	non	61.908

**Table 1:** Part B Patient characteristics; WBC, white blood cell count; PB, peripheral blood; BM, bone marrow; IS, international standard; * no detected *BCR-ABL1* mutation

Patient #	CML Phase	WBC	PB% blasts	BM % blasts	% basophiles	Thrombo cytes	*BCR-ABLI* mutation	IS value
29	CP	57.55	0	3	5	158	D276G	43.43
30	CP	7.36	0	1	0	187	T315I	32.57
31	CP	3.2	0	n.d.	16	167	F317L	35.24
32	CP	235	1	<1	4	565	F359V	48.44
33	CP	8.6	0	n.d.	16	194	F317L	35.1
34	CP	10.3	0	0	3	230	F359I	32.74
35	CP	49.49	0	1	7	1120	G250E	n.d.
36	CP	16.95	1	9	17	111	M244V	n.d.
37	CP	43.7	0	1	13	398	M387L	n.d.
38	CP	37.6	0	1	1	134	V299L	5.681
39	AP	375	5	2	2	351	non	52.79
40	AP	n.d.	n.d.	n.d.	n.d.	n.d.	non	n.d.
41	AP	178.3	7	5	15	626	non	45.389
42	AP	190.9	3	2	15	347	non	48.44
43	AP	145.7	5	n.d.	20	288	non	48.44
44	AP	375	5	2	2	351	non	50.167
45	AP	230	11	3	4	783	non	80.67
46	AP	178.3	7	5	15	626	non	n.d.
47	AP	32.4	1	<1	10	1150	non	42.996
48	AP	46.7	1	3	53	2354	Y253H	n.d.
49	AP	9.56	1	2	1	134	E297K	15.11
50	BC	73.96	44	0	33	33	E255K	44.034

**Figure 1:**
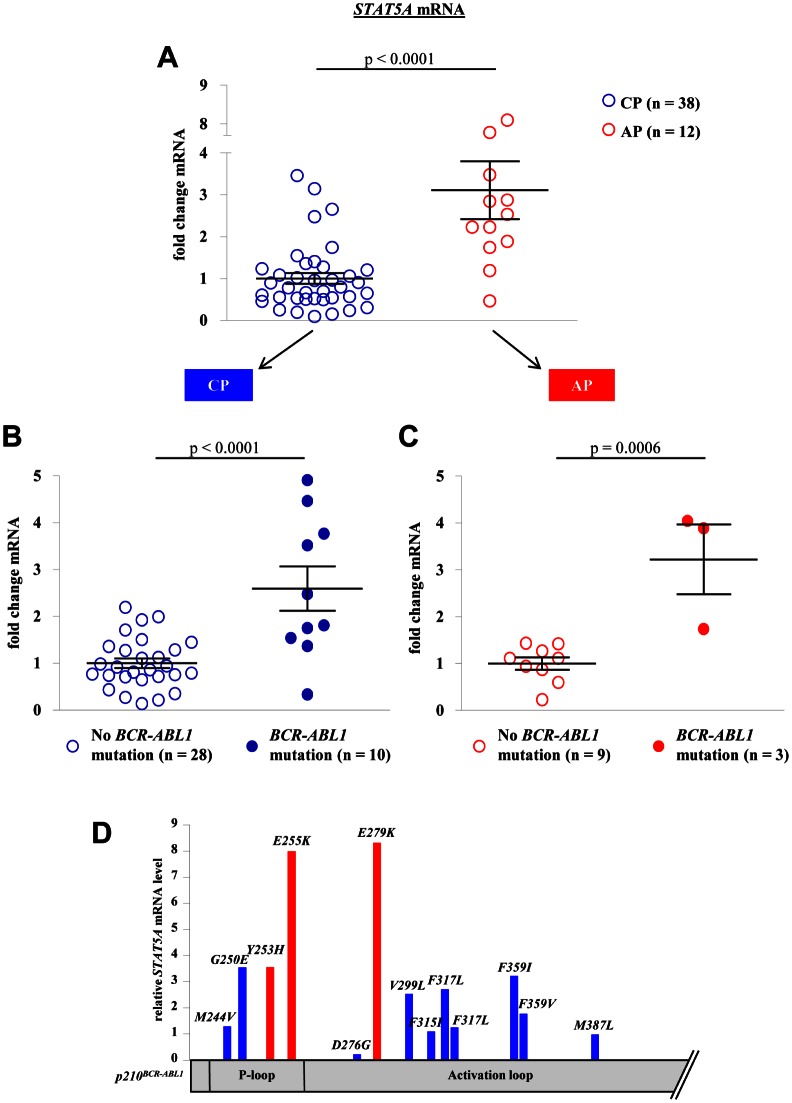
*BCR-ABL1* mutations correlate with *STAT5A* expression levels in primary CML patient samples (A) *STAT5A* mRNA levels of CML-CP patients (n = 38) versus CML-AP patients (n = 12). (B, C) *STAT5A* expression levels of CML-CP (B) and CML-AP (C) patients expressing wild type *BCR-ABL1* versus mutated *BCR-ABL1*. (D) Scheme depicting *BCR-ABL1* amino acid substitutions as well as the relative location on the *BCR-ABL1* gene. The height of each bar represents the corresponding relative *STAT5A* mRNA level.

We confirmed our initial observation that *STAT5A* mRNA expression levels increase during disease progression from CP to AP (p < 0.0001, Fig 1A). Remarkably, the correlation between *STAT5A* expression and *BCR-ABL1* mutations was of high significance (p < 0.0001 and p = 0.0054 for CP and AP, respectively). Patients harboring a mutated form of *BCR-ABL1* displayed elevated levels of *STAT5A* compared to TKI sensitive patients. This correlation was observed in both groups analyzed, in CP- as well as AP-CML patients (Fig 1B and 1C). The distribution of the mutants appears random and not restricted to a distinct set of mutations (Fig 1D).

### Enforced STAT5 expression leads to increased levels of ROS

The correlation between STAT5 expression levels and the frequency of *BCR-ABL1* mutations suggests a causal relationship, which can be tested by exogenous retroviral add back of *Stat5a*. Several mechanisms may link STAT5 to DNA mutations including an increased expression of the PAX5 regulated B-cell mutator protein AID [32], an increased expression of RAD51 DNA-repair proteins [33, 34] or a STAT5 triggered production of ROS [35, 36].

**Figure 2:**
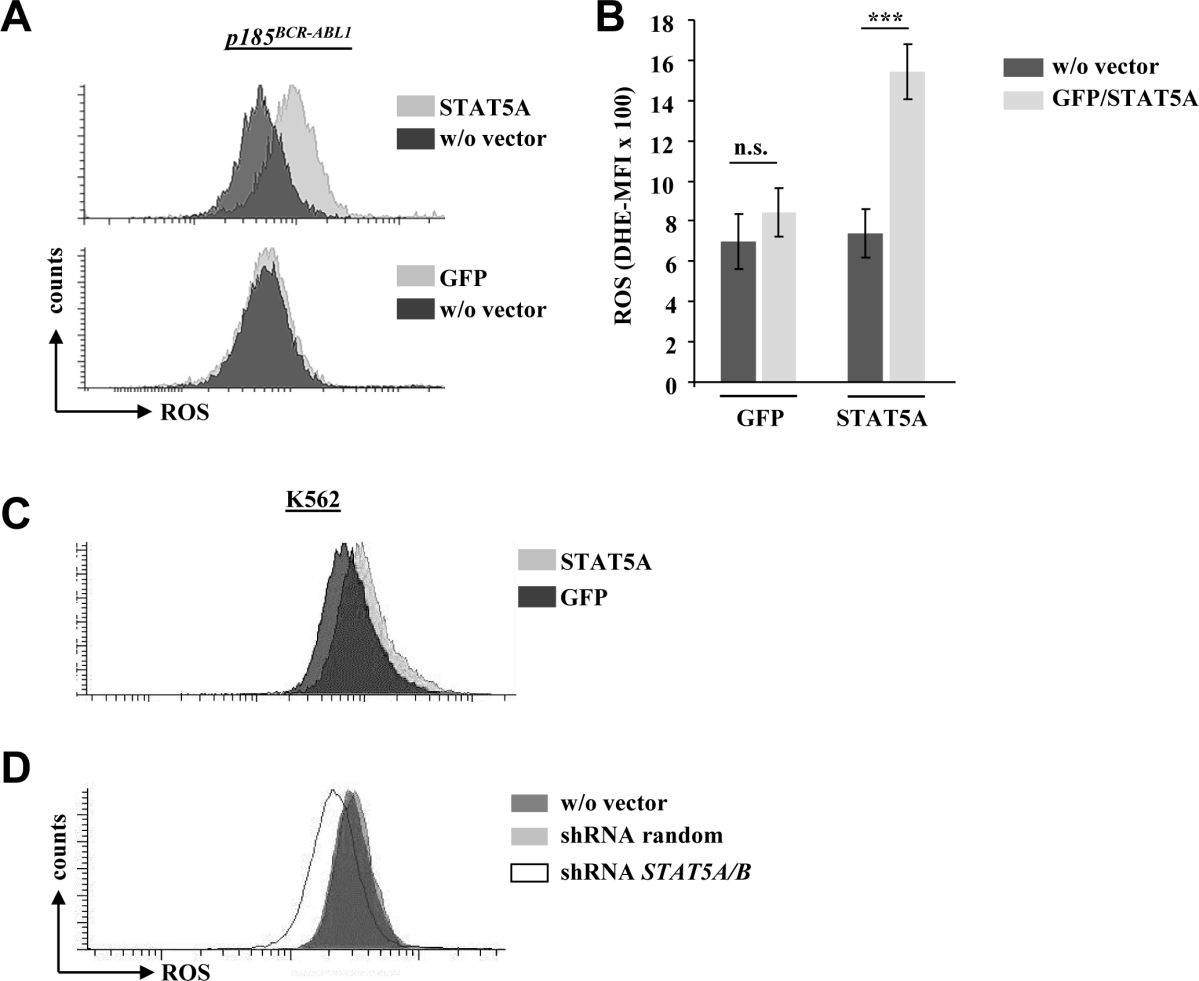
*STAT5A* mediates ROS production in murine and human *BCR-ABL1* transformed cell lines (A) Representative FACS histogram of p185^BCR-ABL1^ transformed murine cells infected with a retrovirus encoding for Stat5a-IRES-GFP (upper histogram) or a control vector (GFP, lower histogram). Cells have been stained with the ROS sensitive fluorescent dye DHE to measure differences in ROS levels upon enforced expression of STAT5A. (B) Statistical analysis showing DHE-MFI of individually derived *p185^BCR-ABL1+^* cell lines (n = 4, data are mean ± SD., n.s. = not significant, *** p < 0.001). (C) FACS histogram depicting ROS level of the human CML cell line K562 upon enforced expression of STAT5A or GFP. (D) K562 cells expressing a random shRNA or a shRNA specific for *STAT5A/B* were stained with DHE to measure differences in ROS levels.

As we failed to detect any changes in PAX5, AID and RAD51 expression in *BCR-ABL1^+^* cells upon enforced STAT5 expression we focused our attention on ROS levels. When we used the ROS sensitive fluorescent dye DHE to stain *p185^BCR-ABL1+^* cells we obtained a clear and consistent picture: the enforced expression of STAT5A - but not the control vector - provoked a significant up-regulation of endogenous ROS levels in 4/4 tested cell lines (Fig 2A and 2B). Comparable results were obtained in human K562 cells upon enforced STAT5A expression (Fig 2C). In line with our findings shRNA mediated down-regulation of STAT5A/B reduced ROS production in K562 cells (Fig 2D). In summary these experiments provided evidence, that STAT5 expression correlates with high ROS levels in *BCR-ABL1^+^* cells.

### STAT5 induced ROS production accompanies enhanced DNA double-strand breaks

**Figure 3:**
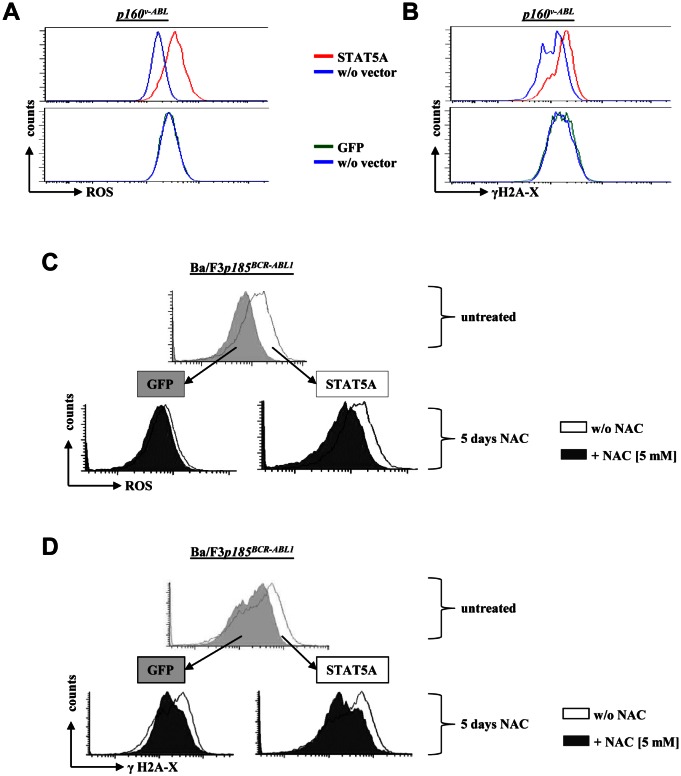
Enforced *STAT5A* expression induces DNA double-strand breaks (A) FACS histogram depicting ROS levels of a p160^v-ABL^ transformed murine cell ectopically expressing STAT5A or GFP. (B) Cells were stained with an intracellular FACS-antibody against γH2A-X to measure differences in DNA double-strand breaks. (C, D) Ba/F3*p185^BCR-ABL1^* cells expressing STAT5A or a control vector (GFP) were treated for five days with the ROS scavenger NAC. Depicted are FACS histograms for (C) ROS and (D) γH2A-X of untreated cells (top of each panel, GFP versus STAT5A expressing cells) and cells treated with NAC compared to controls (bottom of each panel).

ROS is known to induce DNA damage including double-strand brakes (DSBs). Upon DSBs the kinases ATM and/or ATR phosphorylate serine 139 on histone H2A-X (γH2A-X) which quantifies DSBs. We performed intracellular anti-γH2Ax FACS-based staining to test the effects of enforced STAT5 signaling on DNA damage. Indeed, the increased STAT5A mediated ROS levels in *p160^v-ABL+^* cells (Fig 3A) were accompanied by elevated levels of DSBs (Fig 3B). Comparable results were obtained in Ba/F3 cells transformed by p185^BCR-ABL1^. To investigate the potential causal link of STAT5 mediated ROS production and increased DNA-DSB frequency, we used the ROS scavenger N-acetyl-cystein (NAC). Ba/F3*p185^BCR-ABL1^* cells ectopically expressing STAT5A showed an elevated level of ROS compared to control cells that was reduced upon NAC treatment (Fig 3C). In line with this, NAC-mediated decrease in ROS was accompanied by decreased γH2A-X level (Fig 3D).

### STAT5B is a transcriptional regulator of STAT5A

**Figure 4:**
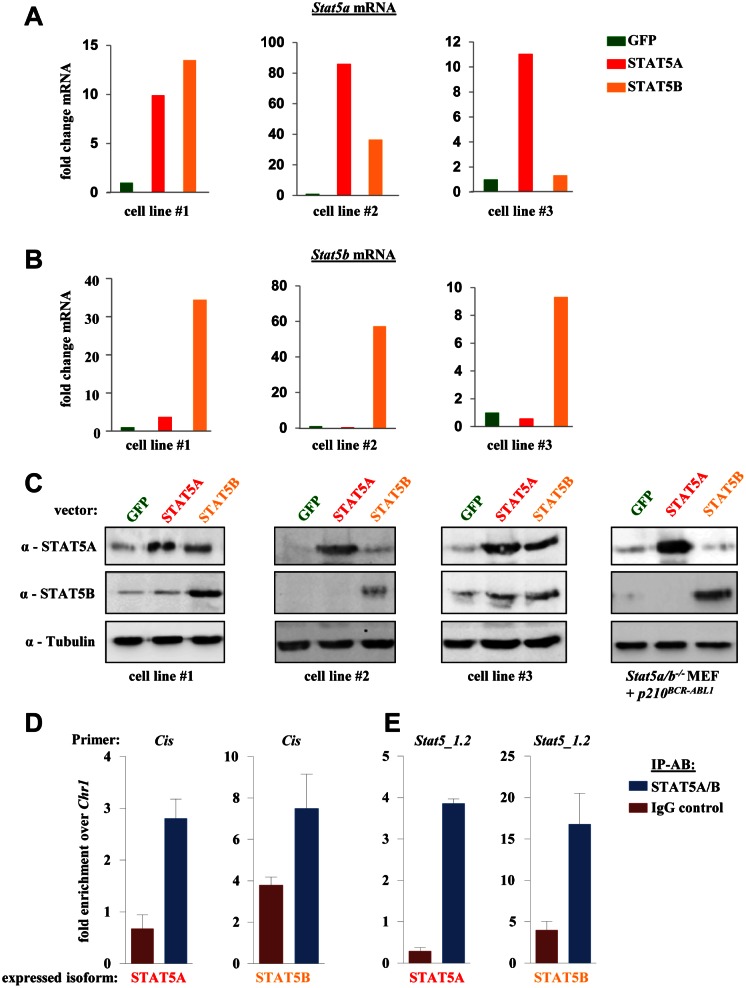
*STAT5B* acts as a transcriptional inducer of STAT5A (A) *Stat5a* and (B) *Stat5b* mRNA levels of three individually derived p160^v-ABL^ transformed murine cell lines ectopically expressing GFP, STAT5A or STAT5B. The fold change in mRNA levels upon enforced STAT5 expression compared to GFP expressing cells is depicted. (C) Immunoblot for STAT5A and STAT5B of three distinct *p160^v-ABL+^* cell lines expressing indicated vectors. *Stat5a/b^−/−^* mouse embryonic fibroblasts ectopically expressing GFP, STAT5A or STAT5 were used as antibody control (right blot). (E, F) ChIP assays were performed using p160^v-ABL^ transformed cell lines ectopically expressing STAT5A or STAT5B. Protein-DNA complexes were immunoprecipitated using antibodies directed against STAT5A/B or IgG (negative control) and analysed by RT-PCR for the presence of (E) *Cis* (positive control) and (F) the *Stat5a* promoter region *Stat5_1.2*. Bar graphs depict fold enrichment over *Chr1*. Experiments have been performed in triplicates.

The *Stat5* gene locus encodes for two distinct but closely related STAT5 isoforms; STAT5A and STAT5B have 97% amino acid homology and fulfill largely redundant and overlapping functions. Differences are present within the extreme C-terminus that account for distinct functions within some cell types. We asked whether the ability to induce ROS production is restricted to STAT5A. To answer this question we generated p160^v-ABL^ transformed murine cell lines and infected them with a retrovirus encoding for STAT5B. RT-PCRs for *Stat5a* and *Stat5b* mRNA were performed to analyze the differences in the expression levels of the distinct *Stat5* isoforms. To our surprise, the enforced expression of *Stat5b* also increased the levels of endogenous *Stat5a* in 2/3 analyzed cell lines (Fig 4A). The *vice versa* effect was not detected and enforced expression of *Stat5a* had no impact on expression levels of endogenous *Stat5b* mRNA (Fig 4B). Enhanced STAT5A protein levels were also detected in several cell lines transduced with a *Stat5b* retrovirus. However, the protein expression levels varied when cell lines were compared on different time points and culture conditions. Representative immunoblots of three distinct cell lines are shown in Fig 4C. Mouse embryonic fibroblasts that lack the *Stat5a/b* gene locus ectopically expressing STAT5A, STAT5B or GFP served as control. Based on these data we analysed a potential transcriptional regulation of *Stat5a* by STAT5B via ChIP. Three conserved candidate sites (*Stat5_1* to *Stat5_3*) in the promoter region of STAT5A/B were chosen based on the consensus sequence of STAT5 (TTCN_3_GAA) (Supporting Information Fig S2A and S2B). *Stat5_1* contains two adjacent conserved sites of which the second one *(Stat5_1.2*) showed prominent binding in ChIP experiments and this site was used for further analysis. A *p160^v-ABL+^* cell line harboring low endogenous STAT5A/B levels ectopically expressing STAT5A or STAT5B was used for ChIP analysis. RT-PCR primers for *Cis* were used as positive control (Fig 4D). For primers amplifying region *Stat5_1.2* we could detect a clear enrichment over *Chr1* (negative control) for both cell lines analyzed (Fig 4E). These ChIP data support our initial findings and indicate a binding of STAT5 in its own promoter region.

### STAT5A and STAT5B increase ROS levels in vivo

**Figure 5:**
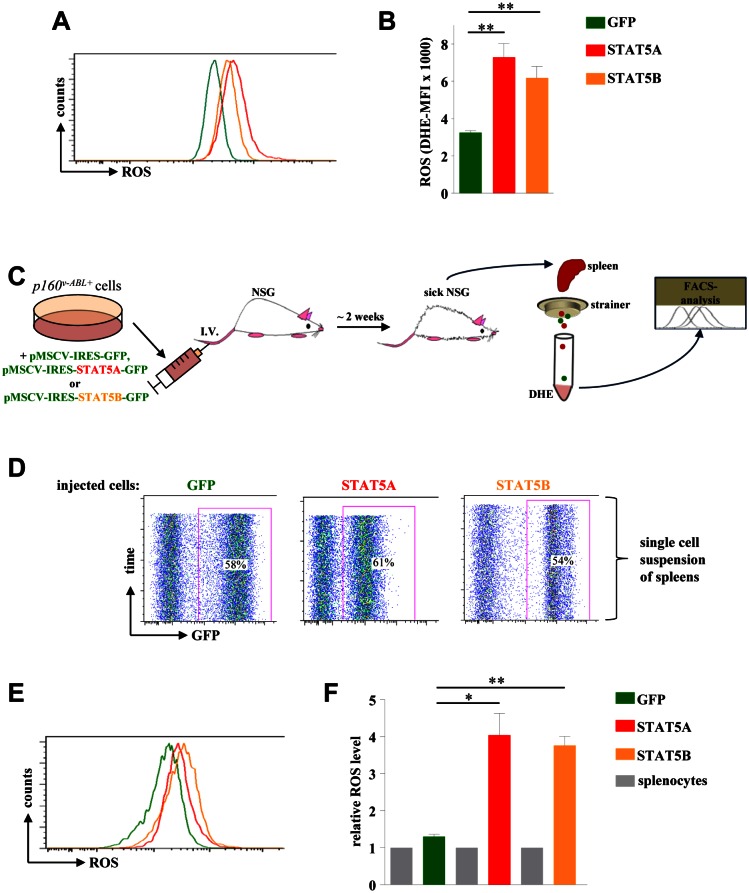
STAT5A and STAT5B expression mediates ROS production in a leukaemia mouse model (A) p160^v-ABL^ transformed murine cells were infected with a retrovirus encoding for GFP, STAT5A or STAT5B and subsequently used to measure ROS levels via DHE staining. (B) Statistical analysis showing DHE-MFI of individually derived *p160^v-ABL+^* cell lines (n = 3). (C) Scheme of experimental procedure used to analyse ROS production of Abelson transformed cells in an in vivo mouse model for leukaemia. (D) FACS blots of single cell suspensions derived from the spleens of diseased mice. The percentage of leukemic cells infiltrating the spleen is shown within the blot. (E) FACS histogram of ex vivo derived leukemic cells expressing GFP, STAT5A or STAT5B stained with DHE to analyse differences in ROS levels. (F) Relative ROS level of ex vivo derived leukemic cells. The fold change in ROS (DHE-MFI) compared to GFP-negative splenocytes is depicted (n = 3). (B and F) Bar graphs represent mean ± SD. * p < 0.05, ** p < 0.01.

As depicted in Fig 5A and 5B both isoforms – STAT5A and STAT5B - were capable to enhance ROS production in Abelson transformed cells which was again accompanied by elevated levels of γH2A-X (Supporting Information Fig S3A). Based on our data presented above indicating a transcriptional regulation of *Stat5a* by STAT5B we cannot completely exclude that the mechanism of STAT5B to induce ROS production is partly through up-regulation of endogenous STAT5A.

One may argue that the enhanced ROS production lacks disease relevance as it may be antagonized in vivo by the microenvironment. To test this hypothesis we intravenously injected p160^vABL^ transformed murine cells ectopically expressing STAT5A or STAT5B as well as control cells into recipient NSG mice. 12 days after injection the mice were sacrificed, spleens isolated, and single cell suspensions were immediately stained with DHE (Fig 5C). The spleens of diseased mice showed infiltration rates of GFP^+^ leukemic cells in the range of 50 to 60% indicating a successful engraftment (Fig 5D). Although this procedure is considerable more stressful for cells compared to an *in vitro* ROS analysis, a reproducible and significant increase in ROS levels upon expression of STAT5A or STAT5B was observed (Fig 5E, 5F and Supporting Information Fig S3B).

### STAT5 mediated ROS production is independent of iron metabolism, Catalase, MnSOD and the mitochondrial respiratory chain

Intracellular ROS levels are maintained within a tight equilibrium and are subject to several layers of regulation including internal and external factors. Iron represents one prominent factor that interferes with ROS levels and ROS staining. Iron is present in culture media or it is an intracellular intermediate product of heme metabolism. As STAT5 has been reported to interfere with iron metabolism in erythroid cells through CD71 and IRP-2 gene regulation [37, 38] we used the iron chelators desferal (DFO) and diethylene triamine pentaacetic acid (DTPA) to test whether differences in iron metabolism or iron level account for STAT5 induced changes in ROS production. As depicted in Supporting Information Fig S4A we failed to detect any changes in ROS production upon iron chelator pre-treatment.

One way how cells control their ROS levels is by tightly regulating the expression of endogenous ROS detoxifying enzymes and scavengers. Two prominent ROS scavengers are Catalase and MnSOD, both regulated by FOXO3a [39, 40]. Expression of neither of these genes was significantly changed upon enforced expression of STAT5A or STAT5B (Supporting Information Fig S4B and S4C). The slight increase of *Catalase* and *MnSOD* mRNA may rather be indicative for an endogenous negative feedback loop caused by elevated ROS levels. It was also shown that inhibition of the mitochondrial respiratory chain (MRC) by rotenone significantly decreased intracellular ROS levels produced by BCR-ABL1 [20]. Thus, the MRC is an important source for ROS production in BCR-ABL1 transformed cells and prompted us to study the role of mitochondria as source of STAT5 mediated ROS production. To avoid off-target effects of inhibitors of the MRC we made use of electro spin resonance spectroscopy (ESR) analysis in combination with cyclic hydroxyl amines (CMH) and iron chelators. This method detects particularly superoxide radicals (O_2_^•^−) rather than other ROS members like hydroxyl radicals or hydrogen peroxide. No significant differences in the level of O_2_^•^− were measured upon retroviral expression of STAT5A or STAT5B (Supporting Information Fig S4D). However, if MRC was inhibited by antimycin A (maximizing O_2_^•^− from MRC by complex III inhibition) all cell lines displayed significantly increased levels of O_2_^•^−. This observation and the lacking differences under basal conditions suggest that the MRC is not a major source of O_2_^•^− upon overexpression of STAT5A or STAT5B. The exclusion of mitochondrial O_2_^•^− points to alternative sources for ROS production than the MRC. The discrepancy between the results obtained with the ESR/CMH method compared to the DHE-based analysis (total ROS) under basal conditions reflects different sensitivity and selectivity (ESR/CMH: O_2_^•^−, DHE: O_2_^•^−, H_2_O_2_, HO^•^) and provides evidence that STAT5A/B acts preferably on the downstream ROS species (H_2_O_2_, HO^•^) or their detoxification.

### STAT5 mediated ROS production is JAK2 independent but depends on BCR-ABL1 signalling and the N-terminus of STAT5

**Figure 6:**
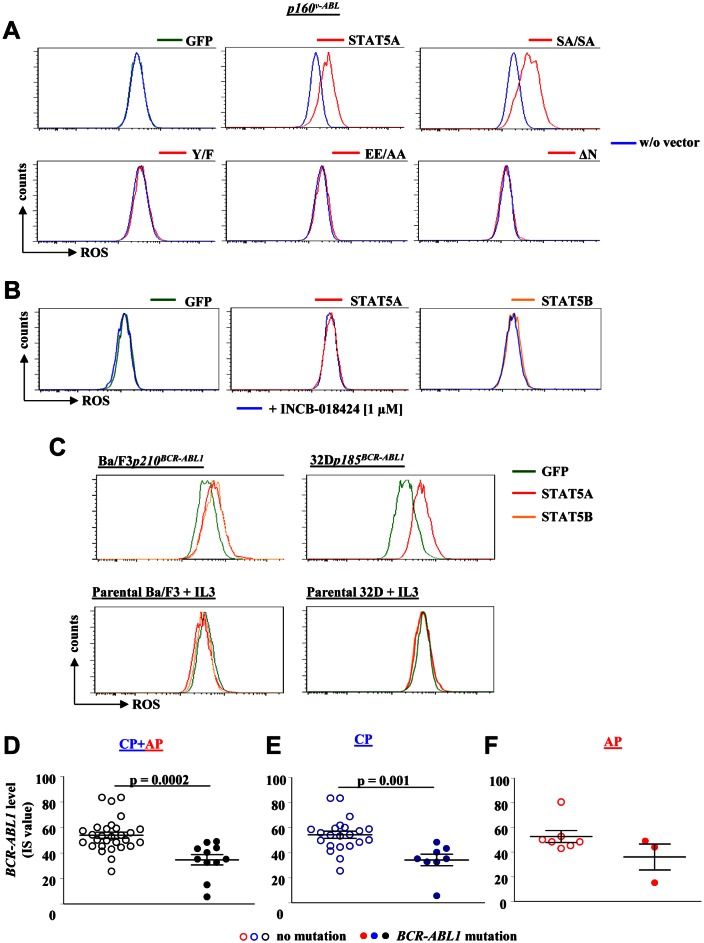
*STAT5* mediated ROS production depends on its N-terminal domain and on BCR-ABL signalling but is independent of JAK2 (A) A *p160^v-ABL+^* cell line ectopically expressing GFP, wild type STAT5A or one of the indicated STAT5A mutants was used to detect differences in ROS level. The experiment has been performed in triplicates. A representative FACS histogram of each cell line is depicted. (B) Two individually derived Abelson transformed cell lines ectopically expressing GFP, STAT5A or STAT5B were treated with the JAK1/2 inhibitor INCB-018424 for 24 hours. Representative histograms of one cell line for each expressed vector treated with INCB-018424 compared to untreated counterparts are depicted. (C) FACS histograms of DHE stained Ba/F3 and 32D cells transformed with p210^BCR-ABL1^ and p185^BCR-ABL1^, respectively (upper panel). The lower panel depicts the ROS level of untransformed parental Ba/F3 and 32D cells growing in IL-3 supplemented medium and ectopically expressing GFP, STAT5A or STAT5B. (D – F) Relative *BCR-ABL1* levels (IS value) of CML patients harbouring wild type *BCR-ABL1* or mutated *BCR-ABL1*. Shown are patients in (D) CP and AP (n = 39), (E) only CP (n = 29) and (F) only AP (n = 10).

Although STAT5 is a classical transcription factor recent evidence revealed a cytoplasmic function for STAT5 in transformed cells. Moreover, we have recently shown that particularly the C-terminal serine sites of STAT5A are needed for efficient myeloid transformation and that both C-terminal serine sites in STAT5A were prominently phosphorylated in CML cells [41]. To gain further insights how STAT5 regulates ROS production, we expressed distinct STAT5A variants in p160^v-ABL^ transformed cells. Besides a GFP-control vector and wild type STAT5A, we expressed a STAT5A variant lacking the serine phosphorylation sites on position 725 and 779 (SASA)[41], an N-terminally truncated STAT5A devoid of the first 136 amino acids (ΔN)[42], a variant not efficiently capable to bind to DNA, but still able to translocate into the nucleus (EE/AA)[43], and a mutant lacking the essential tyrosine phosphorylation site on position 694 (Y/F) [43], devoid of dimerization, efficient nuclear translocation and incapable of DNA binding to STAT5 response elements (for scheme see Supporting Information Fig S5A). STAT5^EE/AA^ and STAT5^Y/F^ failed to increase the level of ROS indicating that the transcriptional activity, dimerization and DNA binding is required for elevating ROS levels. Remarkably, also the STAT5^ΔN^ variant failed to increase ROS levels (Fig 6A). Oligomerization through the STAT5 N-terminus is essential for the transforming potential of constitutively active STAT5 and it was shown to be required for transcription of survival genes or cell cycle regulators [42, 44]. This led us to conclude that an N-terminally regulated target gene might be the trigger for enhanced ROS production.

In hematopoietic non-transformed cells JAK2 is the dominant upstream kinase of STAT5. Although JAK2 is not essential for disease maintenance in CML [45], it still has the potency to phosphorylate STAT5 and is part of a complex with the BCR-ABL1 network [46, 47]. To study the impact of JAK2 for STAT5 mediated ROS production we treated two individual p160^v-ABL^ transformed cell lines expressing GFP, STAT5A or STAT5B with 1 μM of the highly potent and specific JAK1/2 inhibitor INCB-018424 [48] for 24 hours. DHE FACS analysis revealed no impact of JAK2 on cellular ROS levels irrespective whether STAT5 was ectopically expressed or not (Fig 6B).

We asked whether the STAT5 mediated ROS production depends on the presence of the *BCR-ABL1* oncogene. To address this question we made use of parental Ba/F3 and 32D cells growing in IL-3 enriched medium. IL-3 is a potent activator of the JAK2-STAT5 pathway in these cells (Supporting Information Fig S5B-S5D and [45]) thereby giving us the opportunity to compare BCR-ABL1-dependent versus –independent STAT5 mediated ROS production side by side. As expected, Ba/F3*p210^BCR-ABL1^* and 32D*p185^BCR-ABL1^* cells reacted with elevated ROS levels to the enforced expression of STAT5A or STAT5B (Fig 6C, upper panel). Remarkably, the parental Ba/F3 and 32D cells devoid of the *BCR-ABL1* oncogene showed no signs of elevated ROS levels despite enforced STAT5 expression and activation by JAK2 in IL-3 supplemented growth medium (Fig 6C, lower panel). This led us to conclude that oncogenic BCR-ABL1 signalling acts in concerted action with STAT5 to trigger ROS production and to turn STAT5 into a harmful ROS-triggering DNA mutator.

As BCR-ABL1 and STAT5 are both required to increase ROS we analysed the relation between *BCR-ABL1* levels (IS value) and *BCR-ABL1* mutations. To our surprise, we failed to observe a positive correlation. We even detected a slightly negative correlation between IS values and mutation rate (Fig 6D-F) underscoring the importance of STAT5 as mediator of ROS production.

In summary we propose the following model for CML disease progression: In the presence of BCR-ABL1 the increase in STAT5 expression leads to an increased probability to acquire a ROS mediated mutation rendering the *BCR-ABL1* kinase less responsive to TKIs, a well-documented phenomenon of CML progression [49]. The increased mutation rate may provoke apoptosis induction which is effectively counteracted by STAT5 itself as the anti-apoptotic *BCL2* family members *BCL2*, *BCL_XL_*, or *MCL1* are STAT5 target genes as documented by many studies [38, 50-53]. Furthermore STAT5 represses miRNA15/16 that counteracts *BCL-2* and *BCL_XL_* [44]. Therefore, we propose a dual role for STAT5; while increasing the rate of DSBs and therefore *BCR-ABL1* mutation frequency it provides the molecular link to survival gene upregulation to accept elevated DNA damage rate in a subset of leukemic cells with high STAT5 activity (Supporting Information Fig S6A and S6B).

### DISCUSSION

The introduction of imatinib has revolutionized the treatment of CML which has once been a deadly disease before the laboratory of Brian Druker pioneered TKI development. However, the occurrence of BCR-ABL1 TKI resistance limits this success story and additional therapeutic intervention strategies are required. It is noteworthy, that the probability to acquire a *BCR-ABL1* mutation rendering the cells unresponsive to TKIs increases during disease progression from the CP to the more advanced disease stages AP and BCs [49, 54, 55]. In the present study we propose a model how elevated STAT5 expression levels contribute to the enhanced *BCR-ABL1* mutation rates observed in advanced disease phases. The expansion of our cohort of CML patients confirmed our initial finding that STAT5A expression increases during disease progression. It furthermore revealed a highly significant correlation between *STAT5A* mRNA levels and the occurrence of *BCR-ABL1* mutations. As high STAT5 levels in *BCR-ABL1^+^* cells are accompanied by elevated endogenous ROS levels and ROS is a well-known factor responsible for DNA damage and subsequent mutation, we propose a causal link between STAT5 expression, ROS levels and *BCR-ABL1* mutations. The continuous selection pressure subsequently exerted by TKI therapy might select for mutations conferring TKI resistance under a situation where enhanced STAT5 levels and activity promotes survival in a subset of CML clones.

ROS are generated by different sources including NADPH oxidases [56],[57], mitochondria [58], xanthine oxidase [59], as well as endothelial nitric oxide synthase under specific conditions [60]. ROS primarily serves as mediator of important processes including differentiation, host defence [61], oxygen sensing [62], proliferation, apoptosis, and response to mechanical strain [63]. ROS is considered an important signalling mediator [64] and may interfere with gene regulatory pathways such as mitogen-activated protein kinase [65, 66] or hypoxia-responsive-element via hypoxia-inducible factor [67, 68], also known to be regulated by STAT5 transcription factors [69]. Deregulation of ROS metabolism has been implicated in several patho-physiological conditions and has been associated with inflammation, vascular atherosclerosis [70], diabetes [71], hypertension [72], and tumourigenesis including CML [73].

Recent evidence pinpointed at a key role for the Rac2 GTPase in BCR-ABL1 driven ROS production. Rac GTPase alter mitochondrial membrane potentials and electron flow through the mitochondrial respiratory chain complex III (MRC-cIII) in CML cells, thereby generating significant ROS levels [74]. There is at least one additional mechanism how BCR-ABL1 enhances ROS as STAT5 clearly acts via an independent mechanism; electro spin resonance analysis excluded mitochondria as a source of STAT5 triggered ROS production. Of all alternative sources for ROS, NADPH oxidases are of particular interest as NOX4 NADPH has recently been identified as direct transcriptional target of STAT5 in liver and fibroblast cells [75]. All our attempts to detect NOX4 in BCR-ABL1 transformed cells failed. Tissue specific differences in NOX4 expression are the most likely reason underlying this phenomenon. Although we currently cannot identify one or more distinct target genes, we could show that STAT5 induced ROS production require the transcriptional activity of STAT5. Using several STAT5 variants we predict the involvement of the N-terminus which is essential for tetramer formation and the regulation of survival genes (Cite again Li et al., Blood, 2010). Experiments employing STAT5A versus STAT5B revealed that STAT5 is also capable to regulate its own transcription which may at least partially account for the up-regulation of STAT5 protein and mRNA levels observed in CML-AP. Despite high homology between STAT5A and STAT5B pronounced differences are present within the C-terminal transactivation domain of STAT5 isoforms, which are also known to undergo differential post-translational modifications (such as serine phosphorylation, methylation, acetylation), or splicing. Differential STAT5 activities may account for different binding partners and thereby may lead to different sets of target gene regulation.

STAT5 alone does not suffice to regulate ROS but requires the concomitant presence of the *BCR-ABL1* oncogene. This phenomenon is evident from experiments showing that STAT5 fails to increase ROS in Ba/F3 and 32D cells lacking *BCR-ABL*. Despite culture of these parental cells in IL-3 enriched medium leading to constitutive JAK2-STAT5 signalling, the enforced expression of STAT5A or STAT5B did not impact on ROS levels. Several reasons may account for this discrepancy; STAT5 may have a different set of target genes due to distinct co-factors in *BCR-ABL1^+^* cells compared to non-transformed hematopoietic cells. Alternatively, one may envision that decreases in ROS scavenger pathways occur downstream of BCR-ABL1 expression that act in concert with STAT5 to enhance intracellular ROS. In the absence of BCR-ABL1, STAT5 triggered ROS levels in untransformed cells may be “buffered” by scavenger pathways. Along these lines STAT5 may depend on a second signalling pathway activated by BCR-ABL1 to synergistically turn on the ROS-producing machinery. Our results show that STAT5 – albeit harmless to untransformed cells – becomes a potential threat once an oncogene induced signal re-wiring had occurred.

Although the evidence for STAT5 as proto-oncogene is overwhelming one should consider that STAT5 has also been assigned tumour suppressor, differentiation or senescence functions, all counteracting transformation [36, 76]. Constitutively STAT5 (cSTAT5) – induced senescence involves increased production of ROS which accounts for an elevated DNA damage rate linking cSTAT5A expression to p53 induced senescence. These findings again underscore the tight connection between STAT5, ROS and DNA damage [35]. As *BCR-ABL1^+^* cells are largely resistant to senescence induction, CML cells can survive DNA-damage that predisposes them to the development of mutations.

Further protection of *BCR-ABL1^+^* cells stems from the fact that BCR-ABL1-STAT5 not only triggers ROS production but also promotes survival. This protective effect is pronounced in cells expressing high levels of STAT5 as BCL-2, BCL_XL_ and MCL-1 protein levels are under the control of STAT5. This allows the cells to tolerate elevated ROS levels; STAT5 acts in a dual way – while enhancing ROS levels it also provides the cells with the molecular machinery to handle the elevated ROS levels by up-regulating the molecular tools to accept the elevated DNA-damage frequency.

## MATERIALS AND METHODS

### Mice

NOD/Shi-scid/IL-2Rɣ^null^ (NOG) mice were maintained at the Veterinary University of Vienna. NOG mice were used for leukaemia engraftment and ROS ex *vivo* studies. In detail, 5 × 10^5^ cells were injected intravenously and engraftment was monitoring by analysing the percentage of GFP^+^ cells in the peripheral blood of mice. All animal experiments were carried out in accordance with protocols approved by Austrian law.

### Tissue culture conditions and infections

Tissue culture conditions, virus preparation, infection of bone marrow cells with viral supernatant from A010 cells or gp + E86 producer cell lines and establishment of cell lines was described previously [27].

### Immunoblotting

Immunoblots were carried out as described previously.[27, 77] Membranes were probed with antibodies directed against α-tubulin (T9026 Sigma-Aldrich), STAT5A and STAT5B (both described in [78].

### Plasmids

Wild type *Stat5a*, wild type *Stat5b*, the *Stat5a* mutants, *Stat5a^Δ^N* [42], *Stat5a^EE/AA^*, *Stat5a^Y/F^* [43], and the double serine mutant *Stat5a^SS/AA^* [41] were expressed in the retroviral vector *pMSCV-IRES-eGFP*. *p185^BCR-ABL1^* and *p210^BCR-ABL1^* was expressed via a *pMSCV*. Ecotropic, replication incompetent gp + E86 producers were generated and selected for high virus titer production by FACS as described previously [42, 79].

### Primary patient samples

Primary cells were obtained from patients treated at the General Hospital, Vienna, Austria. Cells were obtained from patients with CML at routine blood and bone marrow examinations after informed consent was given. Peripheral blood and bone marrow mononuclear cells were isolated using Ficoll. Samples were analysed for *BCR-ABL1* mutations. Use of human samples was approved by the Ethical Committee of the Medical University of Vienna and is in compliance with Austrian legislation.

### Colony formation and 96-well imatinib resistance assay

For colony formation assays, p160^v-ABL^ transformed cells were plated in cytokine-free methylcellulose (StemCell Technologies) at a density of 2 × 10^2^ cells/ml or 3 × 10^6^ cells/ml without or in the presence of 1 μM imatinib. For the 96-well imatinib resistance assay, 1 × 10^6^ cells/ml were plated in 96-well plates containing RPMI supplemented with 2 μM imatinib. Medium with fresh supplemented imatinib was added every other day. Number of imatinib-resistant clones was evaluated 28 days after the initial imatinib exposure.

### Intracellular pSTAT5 and γH2A-X staining

Cells were analysed by a BD FACS-Canto II FACS device and BD FACS Diva software (Beckton Dickinson). For the intracellular staining 5 × 10^6^ cells were fixed by 2% paraformaldehyde (Aldrich)/PBS at room temperature (RT) for 10 minutes. All washing steps were performed for 15 minutes with 10 ml PBS/2% FCS/0.2% Tween-20 per sample. Cells were washed twice and permeabilized with 99% ice-cold methanol for 20 minutes at −20°C. Cells were washed twice and incubated with αCD16/CD32 (FCγIII/II receptor; BD Bioscience Pharmingen) and 5 μl γH2A-X (phospho histone H2AX S139, Alexa Fluor 647; Cell Signalling) or pSTAT5 (PY-STAT5-Alexa Fluor 647; BD) at RT for one hour. Cells were washed two times before analysing via flow cytometry.

### Real-time PCR

Total RNA was isolated using Tri Reagent (Sigma) according to the manufacturer's instructions. One μg of total RNA was used for cDNA synthesis using the GeneAmp RNA PCR Kit (Roche) and used for the RT-PCR reaction performed on a Biorad MyiQ2 (Biorad) using Sso Fast Eva Green Supermix (Biorad). All experiments were performed in triplicates and repeated at least twice. Sequences of primer pairs used during the course of the study (5'-3'): human *STAT5A* for: GGCTCCCTATAACATGTACCC, rev: AAGACTGTCCATTGGTCGGCG; human *GAPDH* for: TCTCCTCTGACTTCAACAGCG; rev: ACCACCCTGTTGCTGTAGCC; mouse *Bcl2* for: ACTGAGTACCTGAACCGGCATC, rev: GGAGAAATCAAACAGAGGTCGC; mouse *GAPDH* for: AGAAGGTGGTGAAGCAGGCATC, rev: CGGCATCGAAGGTGGAAGAGTG; mouse *MnSOD* for: TTAACGCGCAGATCATGCA, rev: GGTGGCGTTGAGATTGTTCA; mouse *Catalase* for: TGAGAAGCCTAAGAACGCAATTC, rev: CCCTTCGCAGCCATGTG; mouse *Stat5a* for: ACATGGACCAGGCTCCTTCCC, rev: CTCATCCAGGTCAAACTCGCC; mouse *Stat5b* for: GGCAGGGTCAGTAACGGAAG, rev: GGCTCTGCAAAGGCGTTGTC.

### Small interfering RNA mediated knockdown of STAT5A/B

For knockdown of STAT5, an oligonucleotide targeting human and murine *STAT5A* and *STAT5B* mRNA [80] (5'-GCAGCAGACCATCATCCTG-3') was cloned into a modified pLKO.1 lentiviral vector expressing mCherry as a marker. Recombinant VSV-G pseudotyped lentiviruses were produced as described previousely [81]. K562 cells were transduced in the presence of polybrene (7 μg/ml) and knockdown of STAT5 was confirmed by immunoblotting.

### ROS staining

Two days before the staining procedure growth medium was changed and cells were splitted. Only cell lines with less than 10% of dead or apoptotic cells on the day of staining were used. 5 × 10^5^ cells were washed and stained in 4 μM dihydroethidium (DHE; MGT Inc.)/PBS solution for 20 minutes at 37°C. Cells were washed once and immediately used for FACS analysis.

### ChIP analysis

ChIP analyses were performed as described in [82]. For 20 × 10^6^ cells, 5 μg of the respective antibodies were used. For STAT5, a human/mouse STAT5A/B pan specific antibody from R&D systems was used. For IgG isotype control an antibody from Santa Cruz (sc-2027) was used. Conserved STAT5 binding sites were obtained by analyzing sequence conservation between the human and murine *Stat5* locus using the ECR browser (http://ecrbrowser.dcode.org). Putative STAT5 binding sites were identified employing MULTITF (http://rvista.dcode.org). The precipitated DNA was quantified by real-time PCR with a MyiQ instrument (Bio-Rad) [83]. RT-PCR primers: *Chr.1* for: CATAGATGAAGCTGCCACATAGGT, rev: GTGGGCAAGGACAAAGCATTA; Stat5_1.2 for: TCCCTCCCATCCCTCTATTC, rev: AAGCCCCCTTTCCATCTCT; *Cis* for: GTCCAAAGCACTAGACGCCTG, rev: TTCCCGGAAGCCTCATCTT [84].

### Electron spin resonance spectroscopy (ESR)

Cells (6 - 32 Mio. cells/ml) were centrifuged and pellets were resuspended in PBS buffer containing 100 μM DFO (desferal) and 25 μM DTPA (diethylene triamine pentaacetic acid). Cell suspensions were aliquoted and incubated at 30 or 37°C until measurement. Prior to ESR measurements CMH (1-Hydroxy-2,2,5,5-tetramethyl-pyrrolidine-3-carboxylic acid methyl ester) was added. For each data point 17μl of cell suspension were aspirated into a Teflon tube (0.9 mm ID) and transferred to a flexline dielectric resonator ER4118X-MD5 (Bruker, Rheinstetten) for measurement. ESR measurements were performed using a Bruker EMX spectrometer with the following parameters: microwave frequency 9.686 GHz, modulation frequency 100 kHz, modulation amplitude 1 G, time constant 0.082 sec, center field 3448 G, scan rate 71 G/min, sweep 80 G, sweep time 84 s, receiver gain 2 ×10^4^. Always two consecutive scans were recorded and from the peak-to-peak intensity of the middle line the absolute CM^•^ (3-(Carboxy-methyl)-2,2,5,5-tetramethyl-1-pyrrolidinyloxy) concentrations were obtained by comparison with a calibration curve constructed from different CP^•^ (3-(Carboxy)-2,2,5,5-tetramethyl-1-pyrrolidinyloxy) concentrations.

### Chemical compound treatment

To analyse the impact of JAK2 signalling, cells were treated with 1 μM of the JAK1/2 inhibitor INCB-01842 24 hours before DHE staining. To decrease the level of ROS the ROS scavenger N-acetyl-cystein (NAC) was added to the growth medium at a concentration of 5 mM for five days. To analyse differences in pSTAT5 level, cells were treated with 1 μM INCB-0184224 or 1 μM nilotinib 4 hours prior intracellular FACS staining. To decrease levels of endogenous iron, the iron chelators DFO (desferal, 100 μM) and DTPA (diethylene triamine pentaacetic acid, 25 μM) were added for 4 hours prior to measurements.

### Statistics

Two-tailed Student's *t* tests were used for statistical analysis if not stated otherwise. Difference was considered statistically significant when *P* < 0.05. The data are represented as mean ± SD of the number of the determinations and were analysed by Graph Pad^®^ software (San Diego, CA).

## Supplementary Figures


