# Supplementary material for: STAT5 triggers BCR-ABL1 mutation by mediating ROS production in chronic myeloid leukaemia
Source: Oncotarget. 2012 Dec 31;3(12):1669–87. doi: 10.18632/oncotarget.806 (PMC3681503; doi:10.18632/oncotarget.806)
Supplement: Supplementary file 1 [file oncotarget-03-1669-s001.pdf]

Supporting Information

Supp. Figure S1

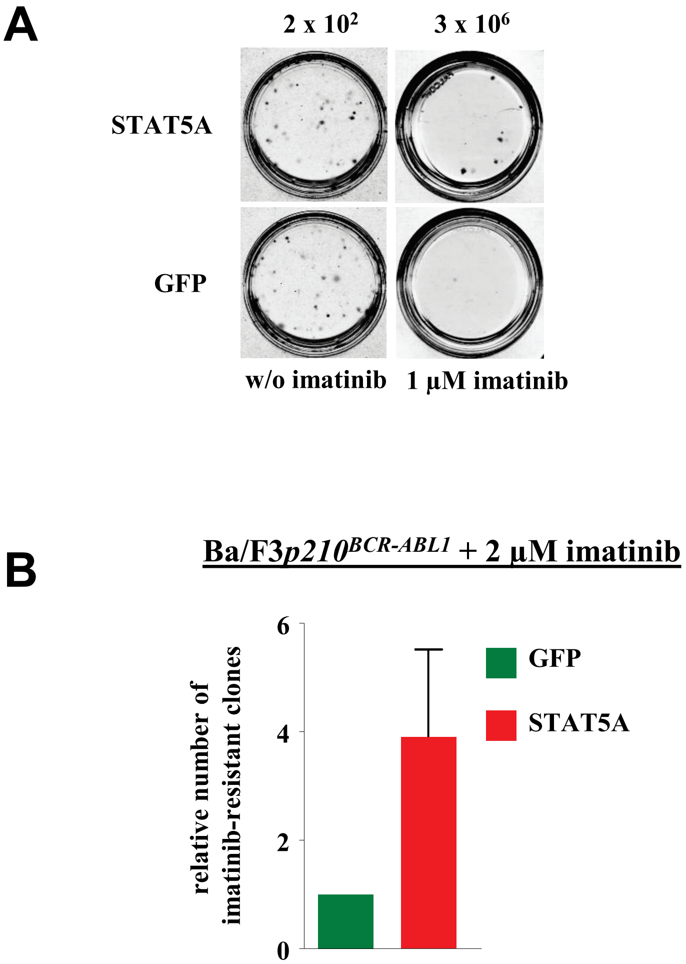

Abelson transformed cell lines overexpressing STAT5A show increased numbers of imatinib resistant clones.

(A) Colony formation assay (CFA) of p160^{v-ABL} transformed murine cells. Indicated numbers of cells ectopically expressing GFP or STAT5A were seeded in the presence of 1 µM imatinib in growth factor free methylcellulose. Untreated cells served as control. Experiments were performed in triplicates and a representative set of data is depicted.

(B) 1 x 10^6 Ba/F3*p210*$^{BCR-ABL1}$ cells/ml were seeded in 96-well plates and treated with 2 µM imatinib. Depicted is the relative number of imatinib-resistant clones ectopically expressing GFP or STAT5A. The experiment has been performed in duplicates. Bar graphs represent mean ± SD.

Supp. Figure S2

A

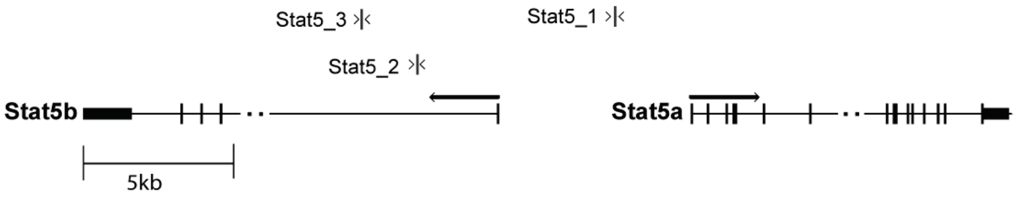

B

```
Stat5_1:   TCTGTAAATTGACTTCTCTGAA (chr11:100,717,481-100,717,502)
Stat5_1.2: TTCCCAGAA (chr11:100,717,415-100,717,423)
Stat5_2:   TTCCCAGAA (chr11:100,708,020-100,708,028)
Stat5_3:   TACAAAGAAATCCCCTGTCAGG (chr11:100,705,377-100,705,398)
```

Binding of STAT5 to conserved *Stat5* consensus sequences within the promoter region.

(A) Genomic location of the *Stat5* locus encompassing *Stat5a* and *Stat5b.* Arrows point to the direction of transcription and boxes depict exons. The location of three conserved STAT5 binding sites is indicated on top (Stat5_1-3). Arrowheads indicate location of primers used for the amplification of immunoprecipitated DNA. Primers binding region STAT5_1 were used for further studies.

(B) Sequences and genomic locations of the conserved STAT5 binding sites. Note that Stat5_1 contains two adjacent conserved sites. Primers binding to the second site (Stat5_1.2) were used for ChIP analysis shown in Figure 4F in the main text.

Supp. Figure S3

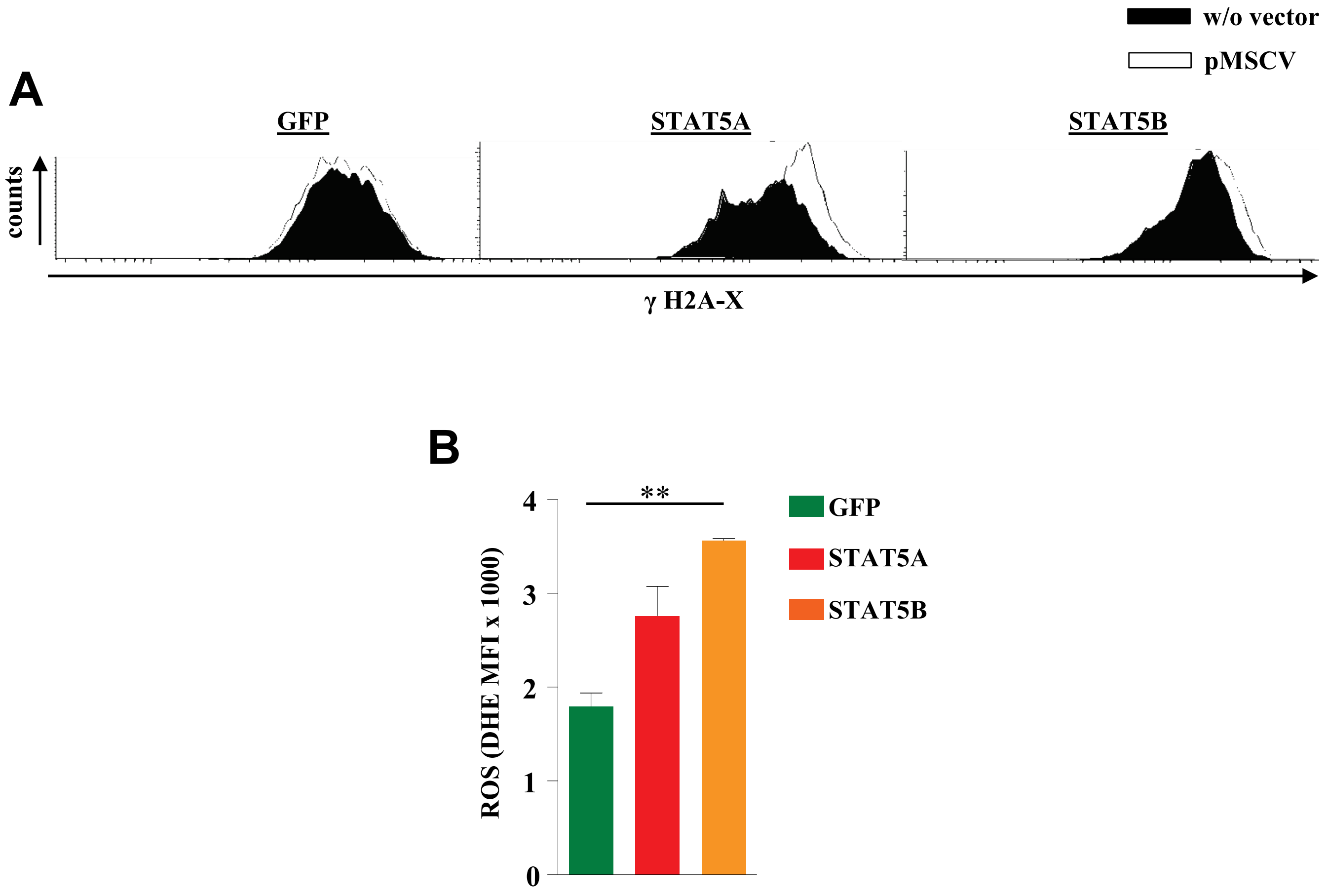

Elevated expression of STAT5A or STAT5B is accompanied by increased levels of DNA-double strand break in vitro and elevated ROS levels in vivo.

(A) FACS histograms for γ H2A-X of p160$^{v\text{-}ABL}$ transformed cells ectopically expressing GFP, STAT5A or STAT5B.

(B) FACS histograms of ex vivo derived leukemic cells expressing GFP, STAT5A or STAT5B stained with DHE were performed to analyse differences in ROS levels. Depicted is the statistical analysis showing DHE-MFI of leukemic cells isolated from the spleen of injected NSG mice (n = 3, ** $p < 0.01$).

Supp. Figure S4

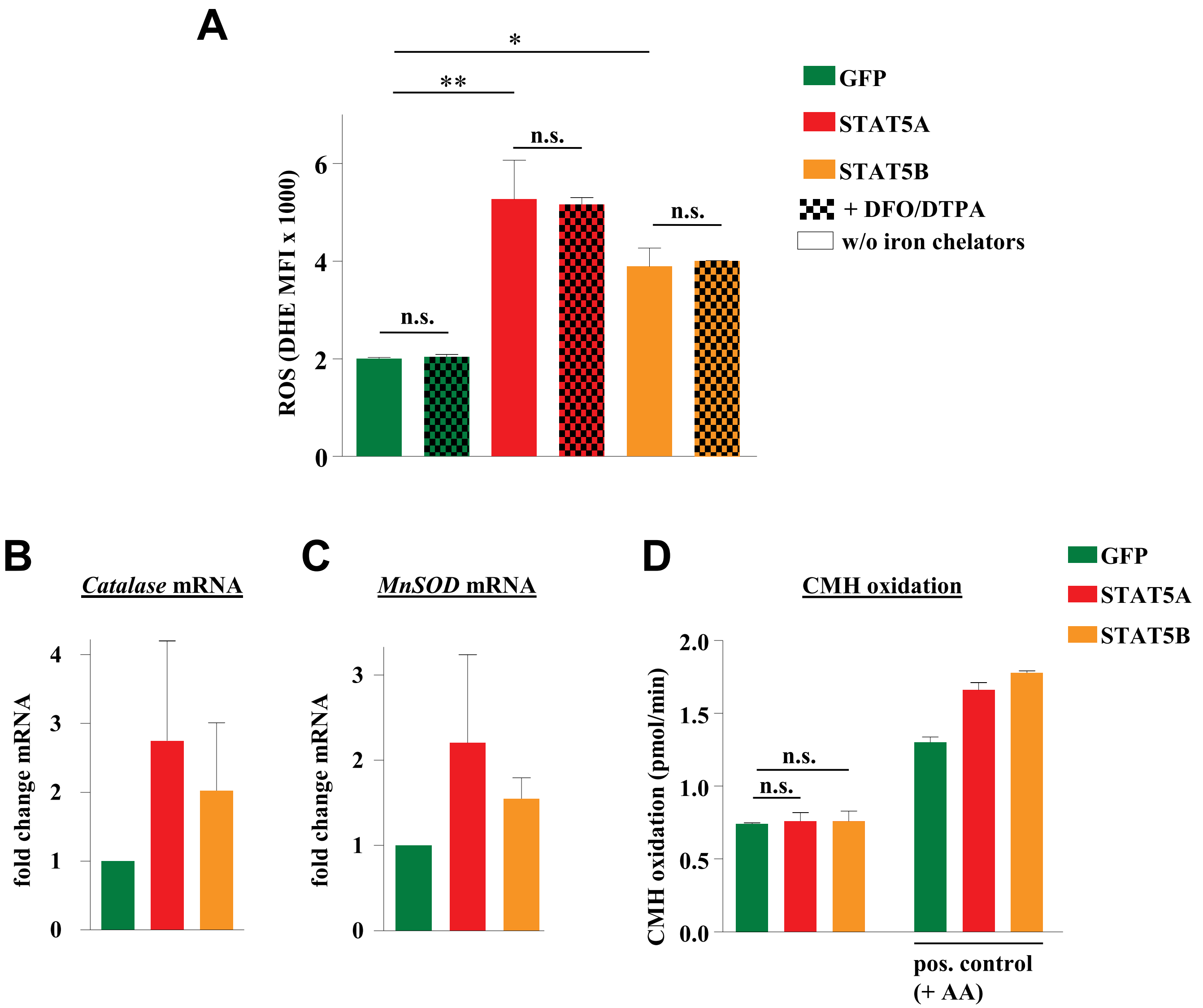

Endogenous iron levels, ROS scavengers and mitochondrial respiratory chain derived ROS are not linked to STAT5 triggered ROS production.

(A) $P160^{v\text{-}ABL}$ ransformed cells were treated with the iron chelators DFO and DTPA for 4 hours prior to staining with DHE. The bar graphs depict ROS levels of cells ectopically expressing GFP, STAT5A or STAT5B with or without DFO/DTPA exposure.

(B) (B, C) RT-PCR for (B) *Catalase* and (C) *MnSOD* mRNA of $p160^{v\text{-}ABL+}$ cells ectopically expressing GFP, STAT5A or STAT5B. The bar graphs depict the fold change in mRNA levels compared to GFP expressing cells.

(C) (D) Bar graphs depict CMH oxidation in pmol/min of Abelson transformed cells expressing GFP, STAT5A or STAT5B. Antimycin A (AA) served as positive control. (A – D) n = 3; bar graphs are mean ± SD.; n.s. = not significant, * $p < 0.05$, ** $p < 0.01$.

Supp. Figure S5

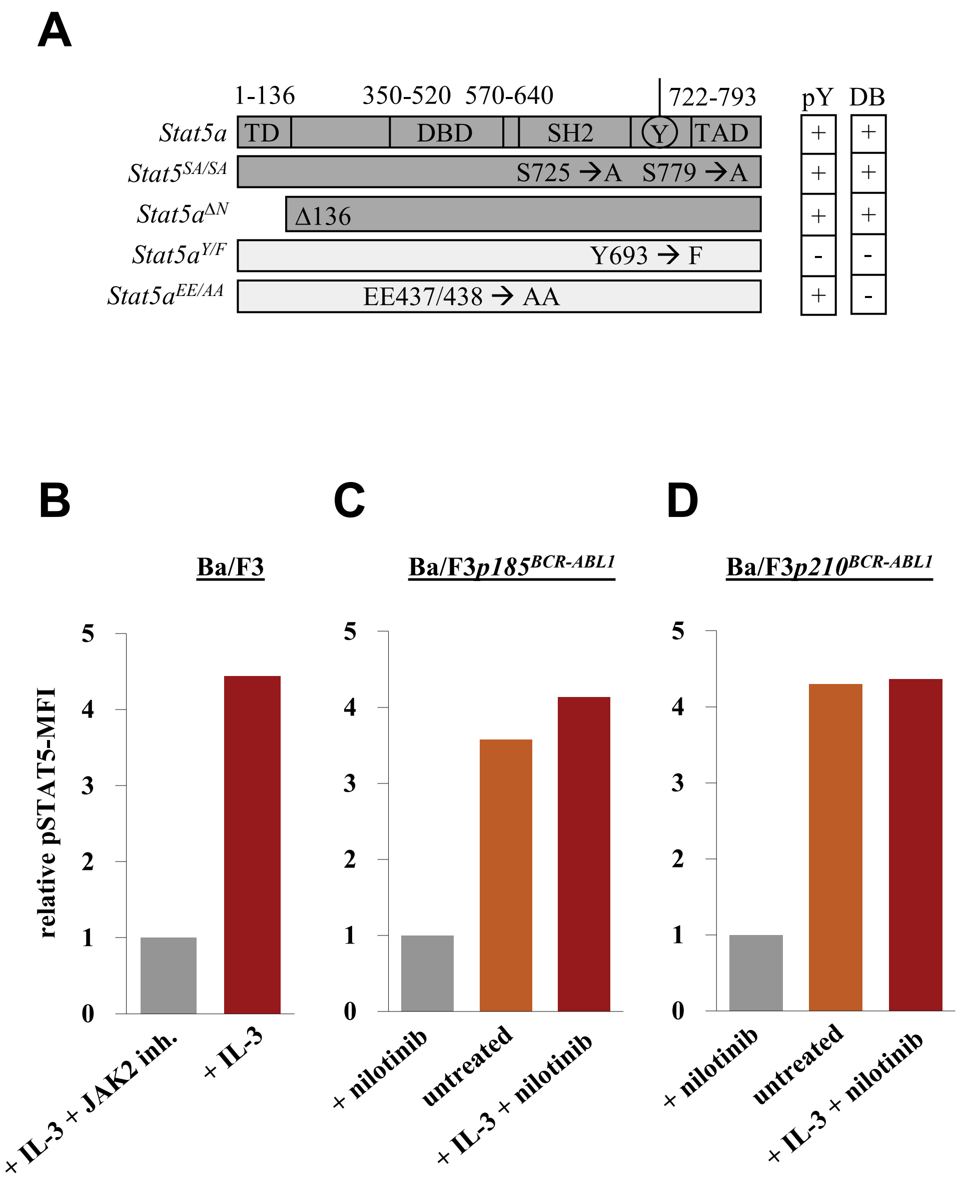

IL-3 is a potent inducer of pSTAT5 signalling in Ba/F3 cells.

(A) Scheme of murine wild-type and mutant *Stat5a* variants. Mutants lacking the tyrosine phosphorylation (pY) site or having impaired DNA binding (DB) are indicated by either (+) or (-).

(B - C) The bar graphs depict the relative pSTAT5-MFI measured via intracellular FACS of (B) parental Ba/F3 cells and Ba/F3 cells transformed with either (C) $p185^{BCR\text{-}ABL1}$ or (D) $p210^{BCR\text{-}ABL1}$. The cells were treated for 4 hours with an JAK2 inhibitor (1 µM) or the BCR-ABL1 inhibitor nilotinib (1 µM). If indicated, IL-3 [2 ng/ml] was supplemented to the medium 24 hours prior fixation of the cells.

Supp. Figure S6

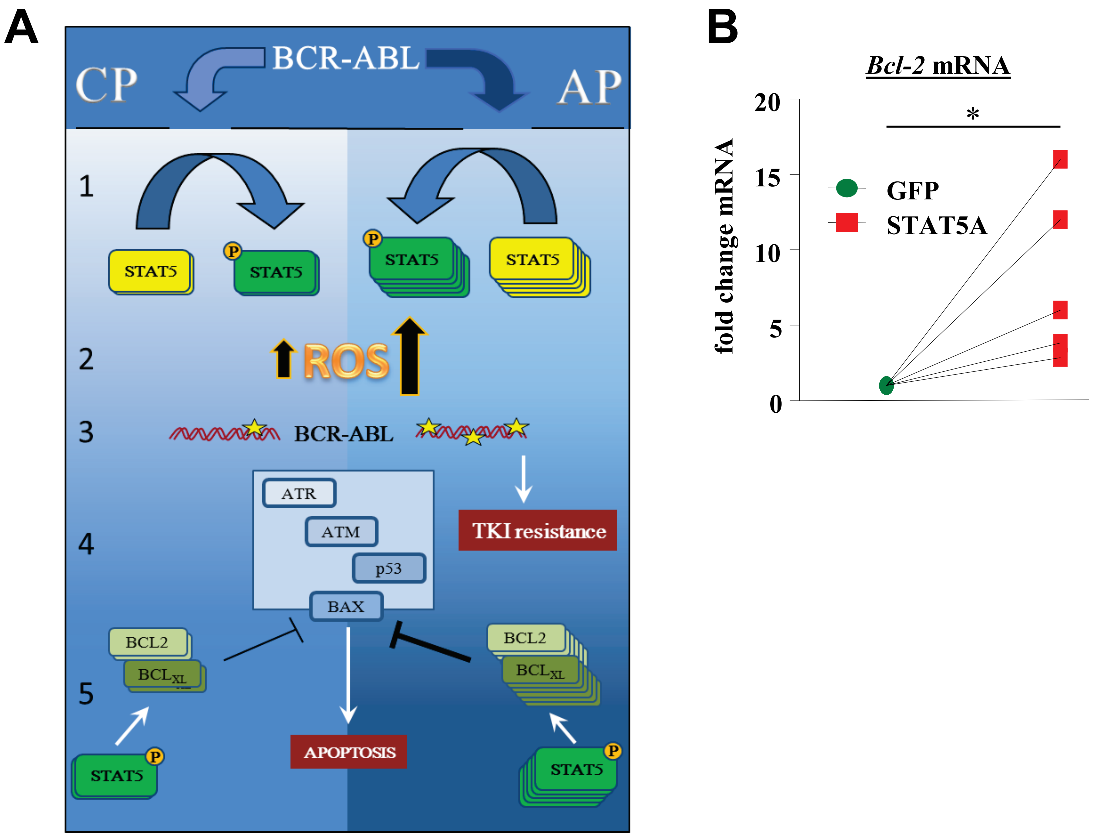

Scheme of signalling pathways downstream of STAT5 contributing to *BCR-ABL1* mutations and increased survival rate in CP (left) and AP (right) cells.

(A) 1) STAT5 becomes phosphorylated and thereby activated by BCR-ABL1. STAT5 expression increases during disease progression from CP to AP. 2) STAT5 mediates ROS production downstream of BCR-ABL1. 3) ROS can induce DNA-damage thereby increasing the probability of BCR-ABL1 to acquire a TKI-resistant mutation. 4) DNA-damage response becomes activated. Depending on the magnitude of DNA-damage pro-apoptotic proteins (e.g. BAX) can induce cell death. 5) This apoptotic pressure can be counteracted by anti-apoptotic proteins like BCL2 or BCL_{XL}; both prominent target genes of STAT5 which are significantly up-regulated upon its activation.

(B) Relative *Bcl2* mRNA level of $p160^{v\text{-}ABL}$ transformed cell lines ectopically expressing GFP or STAT5A ($n = 5$, * $p < 0.05$ with paired t-test).
